# Severe odynophagia in a patient developing after azithromycin intake: a case report

**DOI:** 10.1186/1757-1626-3-48

**Published:** 2010-02-03

**Authors:** Umit Akyuz, Yusuf Erzin, Fevzi Firat Yalniz, Ibrahim Volkan Senkal, Isin Dogan Ekici, Cengiz Pata

**Affiliations:** 1 Department of Gastroenterology, Yeditepe University Medical Faculty, Devlet Yolu Cad 102-104 34752 Kozyatagi, Istanbul, Turkey; 2 Department of Internal Medicine, Yeditepe University Medical Faculty, Devlet Yolu Cad 102-104 34752 Kozyatagi, Istanbul, Turkey; 3 Department of Pathology, Yeditepe University Medical Faculty, Devlet Yolu Cad 102-104 34752 Kozyatagi, Istanbul, Turkey

## Abstract

**Introduction:**

Drug-induced esophageal ulcers most commonly cause heartburn, midsternal pain and dysphagia. In our clinic azithromycin is a relative widely used antibiotic for respiratory tract infections and otitis media because of its activity against Haemophilus influenzae and atypical pathogens, and its ease of administration. After a thorough search in Pubmed the present case is the first one to report azithromycin-induced esophageal ulcer and associated symptoms in the literature.

**Case presentation:**

A 61-year-old Caucasian man was admitted to our endoscopy unit for the investigation of odynophagia and retrosternal pain of new onset. His past medical history was unremarkable but had used azithromycin 500 mg/d for three days in the previous week. An upper endoscopy revealed an extensive serpiginous midesophageal ulcer in the presence of a normal squamocolumnar junction and biopsies from the edges and center of the lesion disclosed no neoplasia or infectious causes but a dense acute inflammatory infiltrate. The patient was put on a liquid diet, sucralfate proton pump inhibitor treatment and was symptom-free within two weeks. After four weeks on therapy a repeated upper endoscopic control examination demonstrated normal findings.

**Conclusion:**

To our knowledge this is the first such a case of azithromycin -induced esophageal ulceration. We think that a little time taken by the physician to warn the patients for taking every oral drug with sufficient amount of water might prevent this kind of complications.

## Introduction

It is well established that various drugs can cause esophageal mucosal injury, tetracycline, doxycycline, quinidine, potassium chloride and emepronium bromide accounting for 90% of reported cases [1]. Severity of injury ranges from mild inflammatory changes to severe ulceration, perforation or stricture formation. Common reason of this complication has been taking medications just before bedtime, and with a small amount of water [1,2]. In this report a patient who developed esophageal ulcers after taking azithromycin (AZM) for upper respiratory tract infection is presented.

## Case presentation

**Figure 1:**
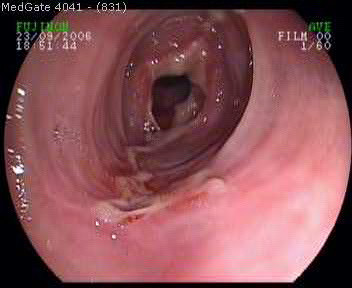
**A serpiginous, in part circumferential mid-esophageal ulcer extending to the distal portion in the presence of normal squamo-columnar junction**.

**Figure 2:**
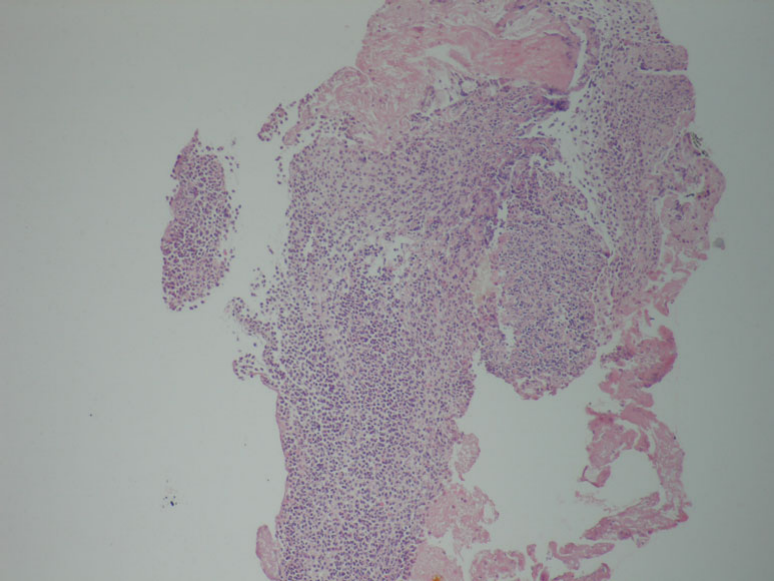
**Dense inflammatory infiltrate around the ulcer base (HE×40)**.

A 61-year-old Caucasian man was admitted to our outpatient clinic with complaints of heartburn, midsternal pain, dysphagia and odynophagia for one week. He was a nonsmoker and denied any use of alcohol, aspirin or non-steroidal anti inflammatory drugs. His past medical history was unremarkable but had used AZM 500 mg/d for three consecutive days after which his complaints began. His physical examination was normal. He had no fever, his cardiac and chest auscultation and throat examinations were normal. An electrocardiography and chest x-ray revealed no pathological findings. An upper endoscopy revealed an extensive serpiginous midesophageal ulcer in the presence of a normal squamocolumnar junction (Figure 1). Biopsies from the edges and center of the lesion disclosed no neoplasia or infectious causes but a dense acute inflammatory infiltrate (Figure 2). The ulceration was thought to be due to AZM intake and the patient immidiately was put on a liquid diet, and sucralfate 1 g. qid, and esomeprazole 40 mg. bid treatment and got symptom-free within two weeks. A control endoscopy demonstrated completely normal findings after four weeks of therapy.

## Discussion

Approximately 100 types of drugs have been incriminated in the etiology of around 1,000 cases of drug-induced esophageal injury (DIEI). The precise mechanism is not well explained. Multiple factors, including the increasing age, decreased esophageal peristalsis and external compression are predisposing to DIEI [3]. But it is noteworthy that among the reported cases of drug-induced injury, the proportion of the patients having a motility disorder such as achalasia and scleroderma or an anatomical narrowing such as tumor or stricture is low [3]. Furthermore, drugs that have a large size and sticky surface are retained longer in the esophagus [4-6]. In addition to these a clinical and experimental study has shown that doxycycline capsules remain three times longer in the esophagus than doxycycline tablets [7].

Esophageal damage mostly occurs at the level of physiological narrowings (aortic arch level or above the lower esophageal sphincter) where the drugs tend to stick [4]. For this reason it seems to be of utmost importance to take high risk drugs with sufficient amount of water.

The most common endoscopic finding is ulcers in varying size, and depth. In very rare cases a stricture formation requireing consequent mechanical dilation may be observed [3]. Esophageal perforation and hemorrhage are rare as well but are life-threatening complications that require immediate specific and aggressive treatment.

The main step of treatment should be the withdrawal of the offending drug. The value of antacids, anti-secretory drug and proton pump inhibitors remain questionable in patients without gastroesophageal reflux [5,7]. Apart from sucralfate, no data from the literature have suggested the benefit of acid suppression [4]. Severe od**y**nophagia rarely requires parenteral hydration or, if prolonged, total parenteral nutrition. Fortunately, in the majority of patients, DIEI symptoms resolve within one week [8].

In conclusion, after an thorough literature search in Pubmed using the keywords AZM and esophagitis, to our knowledge this is the first such a case of AZM -induced esophageal ulceration. We think that a little time taken by the physician to warn the patients for taking every oral drug with sufficient amount of water might prevent this kind of complications.

## Abbreviations

AZM: azithromycin; DIEI: drug-induced esophageal injury

## Consent

Written informed consent was obtained from the patient for publication of this case report and accompanying images. A copy of the written consent is available for review by the Editor-in-Chief of this journal.

## Competing interests

The authors declare that they have no competing interests.

## Authors' contributions

UA performed the upper endoscopy and endoscopic biopsies. YE, FFY, and IVS were major contributors in writing the manuscript. IDE performed the histological examination. CP performed the second look endoscopy.

All authors have read and approved the final manuscript.
